# Mesoporous Silica Nanoparticles for Potential Immunotherapy of Hepatocellular Carcinoma

**DOI:** 10.3389/fbioe.2021.695635

**Published:** 2021-10-08

**Authors:** Han Wu, Xin-Fei Xu, Jia-Qi Zhu, Ming-Da Wang, Chao Li, Lei Liang, Hao Xing, Meng-Chao Wu, Feng Shen, Dong-Sheng Huang, Tian Yang

**Affiliations:** ^1^ Department of Hepatobiliary, Pancreatic and Minimal Invasive Surgery, Zhejiang Provincial People’s Hospital (People’s Hospital of Hangzhou Medical College), Hangzhou, China; ^2^ The Key Laboratory of Tumor Molecular Diagnosis and Individualized Medicine of Zhejiang Province, Zhejiang Provincial People’s Hospital (People’s Hospital of Hangzhou Medical College), Hangzhou, China; ^3^ Department of Hepatobiliary Surgery, Eastern Hepatobiliary Surgery Hospital, Second Military Medical University (Naval Medical University), Shanghai, China; ^4^ School of Clinical Medicine, Hangzhou Medical College, Hangzhou, China

**Keywords:** mesoporous silica, hepatocellular carcinoma, immunotherapy, nanotechnology, review

## Abstract

Hepatocellular carcinoma (HCC) remains a leading cause of cancer-related death worldwide, which lacks effective inhibition of progression and metastasis in the advanced clinical stage. Mesoporous silica nanoparticle (MSN)–based cytotoxic or immunoregulatory drug–loading strategies have attracted widespread attention in the recent years. As a representative of mesoporous biomaterials, MSNs have good biological characteristics and immune activation potential and can cooperate with adjuvants against HCC. This review summarizes the possible future development of the field from the perspective of tumor immunity and aims to stimulate the exploration of the immune mechanism of MSN-based therapy. Through this point of view, we hope to develop new clinical immune drugs that can be applied to HCC clinical management in the future.

## Introduction

Hepatocellular carcinoma (HCC) is the most common pathological type of liver cancer, which remains a great fatal health problem worldwide (Llovet et al., 2021a; Llovet et al., 2021b). Most HCC patients are diagnosed at the advanced stage, and curative methods like hepatic resection or liver transplantation cannot be applied at that time (Chen B. et al., 2020; Rimassa et al., 2021). The first-line management for the advanced HCC contains only systematic therapy such as sorafenib and best supportive therapy according to the BCLC staging system; as a result, more effective methods are urgently needed for clinical management (Bruix et al., 2019; Craig et al., 2020). Present immunotherapy agents like programmed cell death protein 1 (PD-1) blockade regulate the cytotoxic T lymphocytes to attack tumor cells, which are increasingly explored in nano strategies for HCC therapeutic strategies (Cancer Genome Atlas Research Network, 2017; Xu et al., 2019; Sangro et al., 2020; Zheng et al., 2020).

Mesoporous silica nanoparticles (MSNs) have been variously designed to explore the comprehensive tumor diagnosis and treatment due to their high biocompatibility and capability of drug loading in recent years (Nguyen et al., 2019; Liu et al., 2020a; Jiang et al., 2021). Benefiting from the adjustable particle size with a unique shape and specific surface area, MSNs shows great potential in the HCC therapeutic researches (Chen et al., 2018; Li et al., 2018). In order to make better use of the diversified design of MSNs, their surfaces are usually modified and co-assembled with other nanoparticles or molecules for the targeting release at the HCC site (Chen et al., 2015; Wang et al., 2017). Therefore, we try to introduce the design and synthesis of existing multifunctional MSNs and summarize the progressive exploration of MSNs in the direction of HCC immunotherapy.

In this article, the biological and immune effects of MSNs as a multifunctional carrier at the HCC tumor site are discussed, and the applications of MSN tumor vaccine with other physicochemical interventions such as photothermal and hydroxyl radical for immunotherapy are also involved. Such nano-based immune vaccines are verified to enhance the response of the immune system to HCC and allow the body to acquire long-term immunity against tumor recurrence (Le et al., 2019; Song et al., 2019; Yong et al., 2019). Specifically, antigen presentation or immune adjuvant targeting the immune microenvironment can improve the infiltration of effector T cells and enhance the differentiation and infiltration of immune cells to the HCC tumor site. Through analyzing the biological effects of these nanoparticles, we aim to inspire more stable and various designs to apply the potential MSN-loading immunoagents for future HCC clinical management.

## Interaction of Mesoporous Silica Nanoparticles With Immune Microenvironment

### Cellular Uptake and Cargo Release

Injectable MSN solutions are often modified by targeted molecules to increase the chance of endocytosis by tumor cells or macrophages at tumor sites (Li et al., 2010; Meng et al., 2011; Liu et al., 2016). Although monodispersed MSNs can be endocytosis by HCC cells through pinocytosis, they are often unable to avoid lysosomal catalysis and thus reduce the loading drug efficacy. Hyaluronic acid (HA) was conjugated to enhance the targeting effect while protecting the MSNS and its cargo from the lysosomal damage (Zhang et al., 2019). Such effects could be mediated and amplified by CD44 receptor molecules. Ruirui et al. reported that the asialoglycoprotein receptor (ASGPR) was sensitive to lactobionic acid, which was designed to modify the MSNs with pH-sensitive chitosan (Zhao et al., 2017). In this research, ursolic acid and sorafenib were co-loaded for the combined treatment of HCC, which also has great potential in the field of immunotherapy. With its enhanced targeting capability and lysosomal escape, MSNs are more suitable for loading immunogenic agents into tumor regions to play a role in regulating tumor microenvironment (TME).

Compared to traditional drug delivery and release, immune cell uptake with antigen presenting and molecular interaction of MSNs may play an important role in the immune regulation of HCC TME. For example, regulatory T cells could be enriched with miRNA and growth factors released by MSNs, and such effect is often realized through the PI3K/Akt pathway (Liu et al., 2018). Due to the diversity of designing MSNs mesoporous and particle size, different types of drugs such as proteins and nucleic acids can be effectively released. Antigen-presenting cells (APCs) can recognize tumor vaccines carried by MSNs and then activate cytotoxic T cells to exert immune effects (Nguyen et al., 2019). Myeloid-derived suppressor cells (MDSCs) and dendritic cells (DCs) recognize and receive signals carried by MSNs, which often benefit from the exposure and homing of immune cells (Chen G. et al., 2020; Lee et al., 2020; Zuo et al., 2020). As the first batch immune cells exposed to MSNs, the responses of these macrophage subtypes to MSNs are particularly important for the activation of subsequent effector cells, which is also the initiating factor of immunotherapy. MSNs can interact extensively with HCC tumor cells and antigen-presenting cells, promoting targeted drug release and uptake as well as activating immune cells.

### Immune Regulation and Gene Editing

Regulating the state and distribution of immune cells are often the ultimate target mediated by MSN-loading strategies for tumor therapy. Immunogenic cancer cell death (ICD) induced by immunogenic chemo drugs in MSNs could amplify the activation and infiltration of CD8^+^ T lymphocytes (Li et al., 2021). It is very important to induce T lymphocytes to produce effector T cells and prevent the involvement of exhaustive T lymphocytes during the differentiation and clustering of immune cells. Co-delivery of indoleamine-2, 3-dioxygenase (IDO) inhibitors and ICD agents such as doxorubicin could enhance effector T cell infiltration and reduce Treg distribution in several tumor models. In addition, the extra-large pore MSNs delivered the tumor antigen and cytokines to body’s lymph nodes acting as a prophylactic cancer vaccine to activate DC as reported (Cha et al., 2018). In clinical practice, it was often found that immune agents acting alone are ineffective and prone to drug resistance. These co-delivery strategies of MSNs with an immunomodulator or tumor vaccine are effective complements to existing monoclonal antibodies such as PD-1 and CTLA-4, which tend to play an important role in several immune cells and immune organs.

In addition, modulating the phenotype and immune microenvironment of local tumor cells through small interfering RNAs is playing an increasingly frequent role. As similarly mentioned, siVEGF was used as a gene editing tool to modify the tumor microenvironment without affecting normal tissue (Zheng et al., 2018). By identifying downregulated miRNAs, the biological characteristics of MSN can be utilized to modulate the inhibitory effect of the tumor microenvironment (Ahir et al., 2020). Such strategies are expected to function in personalized treatment of HCC tumors to enhance immunotherapy by regulating TME. In addition to miRNAs, cre-recombinase was also packed in MSN loading to edit the gene phenotype of target cells (Martin-Ortigosa et al., 2017). This effective use of genomic enzymes could also be further used in future predictable immunotherapies of HCC. Regulating the immunosuppressive microenvironment and activating relevant immune cells have become the main direction of MSNs participating in tumor immunotherapy.

### Innate Immune Activation and Antigen Presentation

Due to the large specific surface area of the mesoporous structure, MSNs can play a unique role in activating antigen presentation by innate immune cells. For example, MSN-packaged interleukin (IL)-13 in its extra-large-pore could directly activate the macrophages while playing a protective role (Park et al., 2021). MSNs coated by the cancer cell membrane loading the Chinese traditional medicine isoimperatorin could actively target the tumor site, blocking the lymphoma cell cycle and promoting mitochondrial-mediated apoptosis (Zhao et al., 2021). MSNs encapsulating doxorubicin for co-delivering an IDO inhibitor could trigger the release of the IDO inhibitor into tumor extracellular vesicles and also the DOX to the intracellular lysosomal compartment, which were uptaken by IDO-expressing DCs reducing the proportion of immunosuppressive Tregs (Li et al., 2021).

By interacting with antigen presenting cells, MSNs can reverse cellular expression and distribution of immune factors in the immunosuppressive tumor microenvironment (TME). The immuno-MSN was specifically designed for the delivery of cdGMP to activate the stimulator of the interferon gene (STING) for antigen-presenting cells (APCs) in TME (Bielecki et al., 2021). The immuno-MSNs could enhance the recruitment of dendritic cells and tumor-specific macrophages to TME. Inhibition of T lymphocytes can also be reversed by such nano-platforms. Through innate immune activation and antigen presentation, MSNs could prevent the cancer stem cells from escaping immune surveillance (Duan et al., 2021). Under diverse innate immune activation, MSNs can coordinate with a variety of immune cells to regulate TME cytokine expression and reshape the tumor molecular microenvironment.

## Systemic Responses Triggered by Mesoporous Silica Nanoparticles for Potential Hepatocellular Carcinoma Immunotherapy

### Photothermal Amplification on the Immune Effect by Mesoporous Silica Nanoparticles

Photothermal therapy could directly irradiate to the tumor area with the local energy conversion of nanoparticles, which has shown potential in the precise ablation of HCC(Mu et al., 2019; Jędrzak et al., 2020; Zhou et al., 2020). For a long time, researchers focused on the ablation of light and heat, but ignored the immunological effects of doing so. In fact, we suggest that such local damage may have a long-term, systemic effect on the body’s anti-tumor effects although it is not necessarily beneficial. We attempted to summarize the relevant studies on the immunological effects of photothermal stimulation and other *in situ* strategies in Table 1. With the assist of MSNs, photothermal reagents could be transported to the HCC site together with ICD reagents to function *in situ* (Wang et al., 2019). Such ablation may lead to the release of tumor antigens in large quantities, accompanied by the destruction of the HCC cell structure and the necrosis of targeting drugs. The slow release of drugs and energy conversion of MSNS could make the destruction of tumor tissue continue to the whole process of photothermal therapy. There was some evidence that such an effect may modulate the ratio of macrophages, release damage-associated molecular patterns (DAMPs), and cause a subsequent immune cell infiltration (Zhang et al., 2021).

**TABLE 1:** Summary and comparison of MSN-based immune related therapy for HCC.

Adjuvant	Synergistic intervention	Targeting cell lines	Animal model	Immune effector cells	References
IR780	CAR-T cell membrane	SK-HEP-1/Huh 7	BALB/c-nu mice	DCs, CTL	Ma et al. (2020)
Indocyanine green/sorafenib	Photothermal therapy	H22	C57BL/6J mice	DCs, endotheliocyte	Yang et al. (2020)
Sorafenib	Au nanoshell/photothermal	Huh-7/SMMC-7721/HepG2	N/A	Endotheliocyte	Wang et al. (2019)
Doxorubicin	MXene/photothermal	SMMC-7721	Nude mice	DCs, CTL	Li et al. (2018)
Black phosphorus quantum dots	Pt nanoparticles/photodynamic therapy	HepG2	BALB/c nude mice	DCs	Lan et al. (2019)
Sorafenib and CRISPR/Cas9	EGFR gene therapy	HepG2, Huh7	Kunming mice	DCs, endotheliocyte	Zhang et al. (2020)
Diacid metabolite	ABT-737 systematic therapy	H22	Kunming mice	N/A	Liu et al. (2020a)
Irinotecan	N/A	Huh-7	BALB/c nude mice	N/A	Li et al. (2020)
Sorafenib	Ferroptosis	HepG2	N/A	DCs, endotheliocyte	Tang et al. (2019)
HNF4α-encoding plasmid	Cisplatin systematic therapy	Huh7	NOD SCID mice	CSCs	Tsai et al. (2019)
Arsenic trioxide	N/A	H22	BALB/c mice	CTL	Chi et al. (2019)

Abbreviations: MSNs, mesoporous silica nanoparticles; HCC, hepatocellular carcinoma; CAR-T cell, chimeric antigen receptor T cell; DC, dendritic cell; CTL, cytotoxic T lymphocyte; CSCs, cancer stem cells.

In order to further improve the effectiveness of photothermal therapy while amplifying the immune response, higher energy conversion particles need to be sought. Carbon nanodots (CDs) have been used as a good photothermal reagent loading on MSNs due to their unique biocompatibility and tumor site aggregation effect (Qian et al., 2019). Such PTT-induced regulation of NK cells and macrophages can be attempted in a wide range of anticancer fields. The effect of Au NPs on the ablation of HCC is also definite, so the synergistic release of immunomodulatory drugs can be implemented on the basis of existing mesoporous silicon (Li et al., 2019). MSNs loaded with both photothermal agents ICG and DOX could be further incorporated into the poloxamer gel (Fan et al., 2020). This strategy could not only enhance the sustained release of drugs but also enrich the delivery mode of MSNs. The advantage of the mesoporous structure of MSNs is that the drugs that need to be combined can be co-delivered at will only considering compatibility issues.

### Tumor Antigen Presentation With Hydroxyl Radicals

**FIGURE 1:**
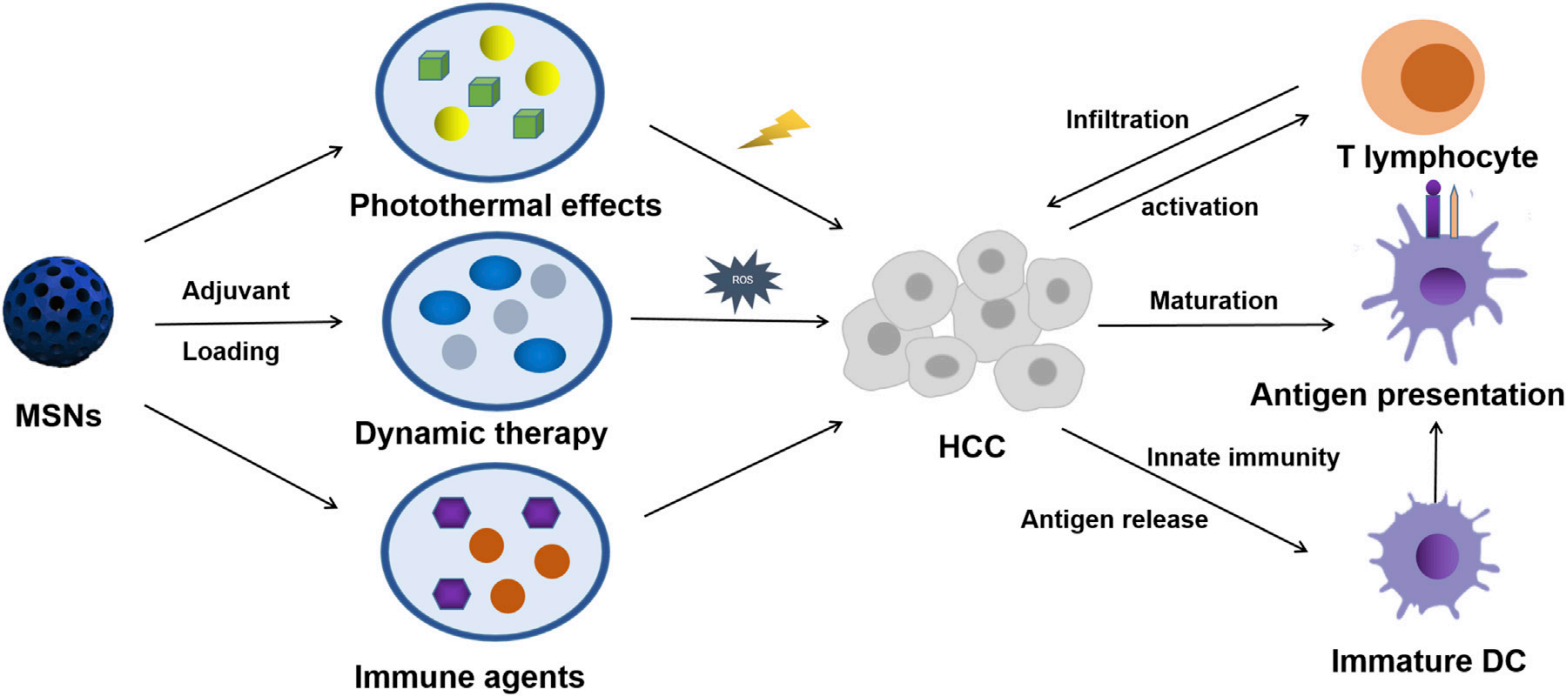
Scheme of MSN-based therapy for triggering HCC systemic antitumor immunological responses.

As already mentioned in Table 1, nanocatcatalysts carried by MSNs were used to generate cytotoxic hydroxyl radicals in tumor sites and have also been widely explored in systematic treatment of solid tumors in recent years (He et al., 2015; Liu et al., 2020b). Such cytotoxic hydroxyl radicals can not only destroy the normal function of organelles, but also more importantly, the damage of reactive oxygen species (ROS) to cell membranes will lead to the presentation of tumor antigens. Although monomorphic MSNs have adverse reactions in liver inflammation mediating and fibrosis, this can provide a better idea for modified MSNs to stimulate tumor inflammation (Zhang et al., 2018; Mahmoud et al., 2019). Local effects of the immune microenvironment, such as ROS on antigen commission, are summarized in Figure 1. The response of the immune system to such an intervention is often diversified, as illustrated in the diagram.

In order to overcome the dependence of single chemokinetic therapy on substrates, photothermal and acoustic dynamic methods could also be used to enhance the efficacy and thus enhance the above local effects (Liu et al., 2020c). And because the catalysts of chemodynamic therapy could often be applied in MR imaging, they are widely used to diagnose the location and progression of tumors (N et al., 2018). Generation of ROS *in situ* therapy overcame the toxic effect of traditional systemic therapy on the normal organs and could be combined with angiogenesis and ICD drugs, which had great potential in the antigen presentation for MSN-based immunotherapy. Similar to the side effects of the cytotoxicity of conventional chemotherapy, the cytotoxic ROS produced by kinetic therapy on the immune system, in particular whether it stimulates HCC progression and metastasis, remains unknown. More studies are needed to understand whether the local killing effect of hydroxyl radicals is a long-term benefit or merely a temporary killer of tumors, and more importantly, what immunological agents need to be used in combination to enhance such long-term effects.

### Synergistic Drug Delivery Based on Immune Antibodies

Due to the lack of effective drugs other than sorafenib in the progression of HCC, many studies have focused on monoclonal antibodies in immunoagents considering their application prospects. Monoclonal antibodies against tumor immunity receptors, such as PD-1 and CTLA-4, have become the focus therapy of various tumors like HCC in recent years (Ruiz de Galarreta et al., 2019; Finn et al., 2020; Kim et al., 2021). Such a strategy can effectively enhance the response of the body’s immune system to tumors and promote the local penetration of immune cells. In fact, despite the lack of studies on direct application of PD-1 in MSN-mediated HCC therapy, MSNs have been shown to be good antibody-carrying and slow-releasing vectors (Sun et al., 2019a; Cheng et al., 2019; Du et al., 2020). The direction of monoclonal antibodies in targeted therapy and the suppression of immune receptors actually depend on the type of the receptor and the distribution of the HCC tumor cell surface. Research on MSNs in this promising area is scarce, which requires researchers to find the right combination strategies while subtly avoiding the side effects of treatment.

The main problems of antibody therapy contain off-target attack of cytotoxic T cells and adaptive desensitization of tumor cells. MSNs can provide a good platform for drug co-delivery of PD-1 and further enhance the sensitivity and specificity of antibodies through other synergistic therapies. For example, inducing the transcription of rRNA to trigger autophagy in tumor cells and disrupting the normal protein transcription and translation process, could be a good complement to immunotherapy (Duo et al., 2018). In addition to playing a role in the tumor sites induced by nanoparticles, antibodies can also help MSNs to isolate and detect circulating tumor cells in the blood (Chang et al., 2018). Such a strategy could well prevent and detect HCC metastasis in the future. In addition, effective immunosurveillance during treatment is also a detail necessary for the application of these antibodies. An overboosted-immune system can sometimes damage normal tissue, and long-term application of the same adjuvant may cause tumor cells to adapt and miss their target.

## Discussion

This mini-review attempts to state the relevant studies of MSNs in HCC immunotherapy, and its advantages are mainly reflected in drug delivery and synergistic multifunctional therapy. As a potential carrier of the tumor nano-vaccine and tumor antigen, MSNs need to be better designed to reduce toxicity and damage to normal tissues. In previous studies, MSNs have shown good efficacy in photothermal therapy, dynamic therapy, and monoclonal antibody therapy and have the potential to be used in combination with immunotherapy (Sun et al., 2019b; Kim et al., 2019; Lee et al., 2019). These studies often lack the exploration of the mechanism of specific immune agents and immune responses and only focus on the direct killing effect of drugs or physical and chemical factors on the tumor itself. Therefore, we not only need to explore the immune mechanism of various cytotoxic drugs, but also need to find new MSN components and immune adjuvants that can be used in immunotherapy.

Although MSNs have the potential to act as immune vectors, there are still some challenges in clinical transformation. First, clinical trials should prove that the basic drug research studies using several medicine are better than the present strategy like PD-1 or CAR-T. Second, both preparation and retention must be taken into account in adapting to diverse hybrid molecular combinations. Finally, stronger evidence of local and systemic immunological reactions is needed to support mechanisms of action in a range of combinatorial applications. As a relatively mature biological carrier, it is not difficult to solve these problems in the application of MSNs in immunity.

Despite the lack of directly relevant evidence in some aspects of HCC immunotherapy, we try to describe the potential applications of MSNs in the aforementioned immune-related field by inducing other necessary studies as mentioned. MSNs may play a greater role in overcoming the side effects of immunotherapy and enhancing the sensitivity of tumor cells to immunotherapy. Most relevant researchers have not yet realized the double-sided characters of local nano-based killing of tumors. The key lies in how to make good use of the cytotoxic drugs carried by nanoparticles while avoiding the stimulation of tumor metastasis, which especially relies on the antigen-presenting cells and memory T lymphocytes. We look forward to more and better works in this area to help clinically manage patients with advanced HCC. At the same time, it may help explore the application of inorganic mesoporous nanoparticles in the field of tumor therapy.
